# Pulmonary Artery Occlusion and Mediastinal Fibrosis in a Patient on Dopamine Agonist Treatment for Hyperprolactinemia

**DOI:** 10.3389/fphar.2017.00492

**Published:** 2017-07-20

**Authors:** Junjing Su, Ulf Simonsen, Jørn Carlsen, Soren Mellemkjaer

**Affiliations:** ^1^Department of Biomedicine and Pharmacology, Aarhus University Aarhus, Denmark; ^2^Department of Cardiology, Rigshospitalet Copenhagen, Denmark; ^3^Department of Cardiology, Aarhus University Hospital Aarhus, Denmark

**Keywords:** dopamine agonist, serotonin, pulmonary hypertension, mediastinal fibrosis, adverse drug reaction

## Abstract

Unusual forms of pulmonary hypertension include pulmonary hypertension related to mediastinal fibrosis and the use of serotonergic drugs. Here, we describe a patient with diffuse mediastinal fibrosis and pulmonary hypertension while she was on dopamine agonist therapy. A young woman, who was treated with cabergoline and bromocriptine for hyperprolactinemia, presented with progressive dyspnea over several months. Based on the clinical investigation results, in particular, elevated pulmonary arterial pressures and significant perfusion defects on computed tomography (CT) pulmonary angiography and ventilation/perfusion (V/Q) scintigraphy, chronic thromboembolic pulmonary hypertension (CTEPH) was initially considered the most plausible diagnosis. However, during an attempted pulmonary endarterectomy, loose fibrous tissues were observed in the mediastinum and cryosection of the right pulmonary artery showed fibrosis and chronic inflammation. Subsequent investigations revealed that diffuse mediastinal fibrosis with concurrent pulmonary hypertension, and not CTEPH, was the most likely diagnosis and cabergoline and bromocriptine may have triggered the fibrotic changes. Both drugs are ergot-derived dopamine agonists, which are known to cause cardiac valve fibrosis and less frequently, non-cardiac fibrotic changes. The underlying mechanism is attributed to their interactions with serotonin receptors. There is much evidence that serotonin, a potent vasoconstrictor and mitogen, is involved in the pathogenesis of pulmonary hypertension. In conclusion, as CT and V/Q scintigraphy findings can occasionally be deceptive, physicians should be particularly aware of differential diagnoses in patients without obvious history of venous thromboembolism that are suspected of having chronic thromboembolic pulmonary hypertension.

## Introduction

Pulmonary hypertension, defined as an increase in resting mean pulmonary arterial pressure (≥ 25 mmHg) as assessed by right heart catheterization, is a serious condition that can lead to right heart failure (Galie et al., 2016). Several forms of pulmonary hypertension exist, including chronic thromboembolic pulmonary hypertension (CTEPH), which is caused by obstruction of the large pulmonary arteries typically following an episode or recurrent episodes of pulmonary embolism. The treatment of choice for CTEPH is pulmonary endarterectomy, which is potentially curative (Jamieson et al., 2003). It is therefore imperative to identify patients that are eligible for surgery.

Other unusual forms of pulmonary hypertension include pulmonary hypertension related to mediastinal fibrosis (Seferian et al., 2015) and the use of serotonergic drugs (Seferian et al., 2013). Mediastinal fibrosis (fibrosing mediastinitis) is a rare condition characterized by proliferation of fibrous tissues in the mediastinum often associated with granulomatous diseases, such as histoplasmosis, tuberculosis and sarcoidosis and other fibro-inflammatory and autoimmune diseases (Rossi et al., 2016). It can be induced iatrogenically in relation to previous mediastinal irradiation and treatment with methysergide maleate (Graham et al., 1966), an ergot-derived serotonin antagonists previously used in the treatment of migraine. Mediastinal fibrosis can cause compression and obliteration of vital mediastinal structures, i.e., the airways, esophagus and great vessels (Sherrick et al., 1994). Thin-walled vessels with low intraluminal pressure, such as superior vena cava and less frequently, the pulmonary arteries, are especially subjected to compression by mediastinal masses resulting in increased intravascular pressure. Due to its extended course through the mediastinum, the right pulmonary artery is more susceptible to mediastinal processes compared to the left pulmonary artery.

Serotonin is a potent vasoconstrictor and mitogen that causes smooth muscle cell hyperplasia and hypertrophy. There is much evidence that it is involved in the pathogenesis of pulmonary hypertension (Egermayer et al., 1999). In addition, an association between the appetite suppressants, fenfluramine derivatives, and pulmonary hypertension is well-established. Fenfluramine derivatives cause increased serotonin levels by acting as serotonin uptake inhibitors and induce transport-mediated serotonin release (Seferian et al., 2013). However, whether other serotonergic drugs, such as ergot-derived dopamine agonists, are associated with an increased risk of developing pulmonary hypertension remains unclear. Here, we describe a patient on ergot-derived dopamine agonist therapy for hyperprolactinemia that had diffuse mediastinal fibrosis and pulmonary hypertension mimicking CTEPH.

## Case Presentation

A 36-year-old woman, who presented with progressive exertional dyspnea over 6 months, was referred to us as a potential candidate for pulmonary endarterectomy with a presumptive diagnosis of CTEPH, for which she was receiving anticoagulation treatment. She did not report any chest pain or episodes of syncope. She was diagnosed with a microprolactinoma after experiencing galactorrhoea 7 years earlier, for which she received low-dose cabergoline treatment (0.5 mg/week) for a year before switching to bromocriptine (2.5 mg/day) when she wished to become pregnant. She took bromocriptine for a year until she became pregnant and the treatment was paused. Shortly after a non-problematic pregnancy and childbirth, low-dose cabergoline treatment (0.5 mg/week) was resumed. In total, the patient had received ∼160 mg cabergoline and ∼900 mg bromocriptine and her prolactin level remained within normal range on treatment. She was a non-smoker and was on birth control pills for several years. The medical history on the father’s side was unknown and there was nothing remarkable on the mother’s side.

**FIGURE 1:**
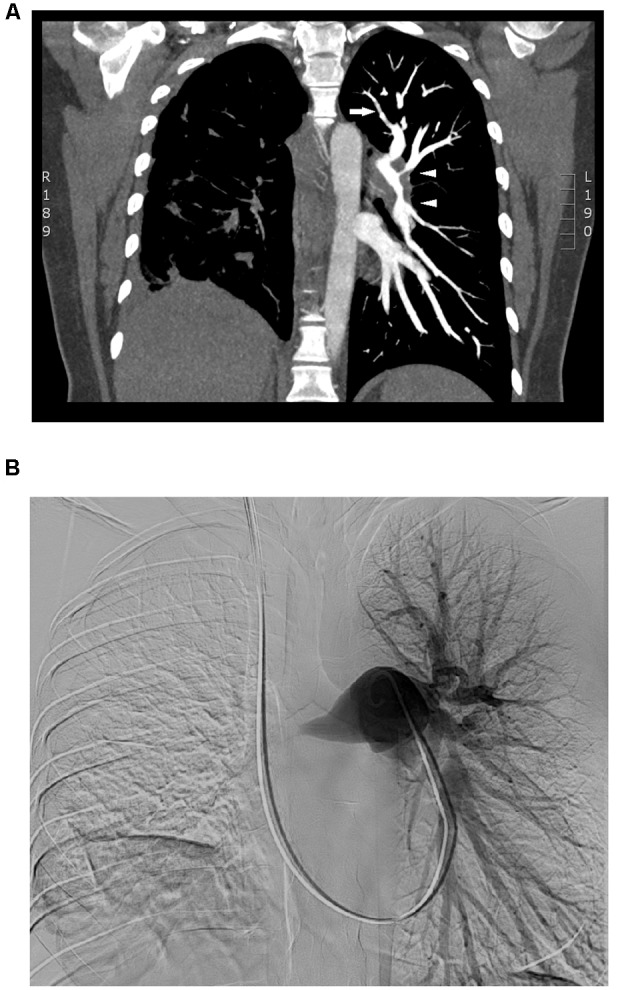
Pulmonary artery occlusion. CT pulmonary angiography **(A)** showed near-complete occlusion of the right pulmonary artery without any flow and elevated right hemidiaphragm. There was severe thickening of the left lobar pulmonary arteries and short-segment stenosis (white triangles) and wall irregularities (white arrow) in the left lobar and segmental pulmonary arteries. Similar findings were observed during digital subtraction angiography **(B)**.

Clinical examination of the patient revealed a systolic murmur and reduced breath sound in the right lung. The 6-min-walk-distance was reduced to 259 m. An electrocardiogram showed T-wave inversion in leads V3 and V4. Creatine kinase MB isoenzyme and troponin T levels were within normal range. However, the level of the NT-proBNP was elevated to 2899 ng/L. Transthoracic echocardiography revealed a moderately dilated right ventricle with impaired function and moderate tricuspid regurgitation with a gradient of 92 mmHg. Subsequent right heart catheterization showed severe pre-capillary pulmonary hypertension with a mean pulmonary arterial pressure of 52 mmHg and pulmonary vascular resistance of 880 dyn.s/cm^5^ (a summary of main findings are listed in **Table 1**). HRCT showed significantly thickened left and right pulmonary arteries, elevated right hemidiaphragm and non-specific infiltrates in the right lower lung. There was no evidence of mediastinal lymphadenopathy. CT pulmonary angiography revealed near-complete occlusion of the right pulmonary artery and severe, short-segment stenosis as well as wall irregularities in the left lobar and segmental pulmonary arteries. Similar findings were observed during digital subtraction angiography (**Figure 1**). PET-CT did not show any pathological FDG-uptake. V/Q scintigraphy revealed absent perfusion to the right lung that was not matched to the ventilation and several segmental and sub-segmental mismatched perfusion defects in the left lung. Spirometry showed reduced lung function of a restrictive pattern. Biochemical and serological screening for thrombophilia, autoimmune diseases and systemic connective tissue diseases were all negative. Based on results from the above-mentioned investigations, CTEPH as a consequence of previous subclinical pulmonary embolism was initially considered the most plausible diagnosis.

**Table 1:** Summary of main findings.

Vital signs	Heart rate: 97 min^-1^, blood pressure: 125/80 mmHg, temperature: 37.6°C, saturation: 95%
6-min walk distance	259 m
Electrocardiography	Sinus rhythm, T-wave inversion in V3 and V4
Transthoracic echocardiography	Normal sized left atrium, normal left ventricular dimensions and function, moderately dilated right atrium and ventricle, TAPSE: 1.4 cm, -moderate tricuspid regurgitation with a gradient of 92 mmHg, dilated IVC with absent respiratory variation
Right heart catheterization	CI (thermodilution): 1.8 L/min/m^2^, right atrial pressure: 19 mmHg, PAPm: 52 mmHg, PAWP: 13 mmHg, PVR: 880 dyn.s/cm^5^
Coronary angiography	Normal
HRCT	Thickened pulmonary arteries, elevated right hemidiaphragm, nonspecific infiltrates in the right lower lung
PET-CT	No pathological FDG-uptake
CTPA and DSA	See **Figure 1**
V/Q scintigraphy	Absent perfusion to the right lung, segmental and sub-segmental mismatched perfusion defects in the left lung
Spirometry	FEV1: 1.36 (42% predicted), FVC: 1.79 (48% predicted), FEV1/FVC: 76%
Histology	See **Figure 2**
Bronchoscopy with EBUS	No evidence of malignant cells or granulomatous inflammation in the endobronchial biopsy, bronchoalveolar lavage fluid and transbronchial needle aspiration biopsy from lower paratracheal and subcarinal lymph nodes.
Blood test	Normal blood count, normal liver and kidney function tests, C-reactive protein: 26 mg/L, erythrocyte sedimentation rate: 14 mm/hr, prolactin: 288 mIU/L (on cabergoline treatment), NT-proBNP: 2899 ng/L, troponin-T: below limit of detection, negative screening for thrombophilia, autoimmune and connective tissue diseases

*Bronchoscopy results were obtained after the attempted pulmonary endarterectomy, while the rest of the results were obtained prior to or during the procedure. CI, cardiac index; CT, computed tomography; CTPA, CT pulmonary angiography; DSA, digital subtraction angiography; EBUS, endobronchial ultrasound; FDG, fluorodeoxyglucose; FEV1, forced expiratory volume in the first second; FVC, forced vital capacity; HRCT, high resolution CT; IVC, inferior vena cava; NT-proBNP, terminal pro-B-type natriuretic peptide; PAPm, mean pulmonary arterial pressure; PAWP, pulmonary arterial wedge pressure; PVR, pulmonary vascular resistance; PET, positive emission tomography; TAPSE, tricuspid annular plane systolic excursion; V/Q, ventilation/perfusion*.

**FIGURE 2:**
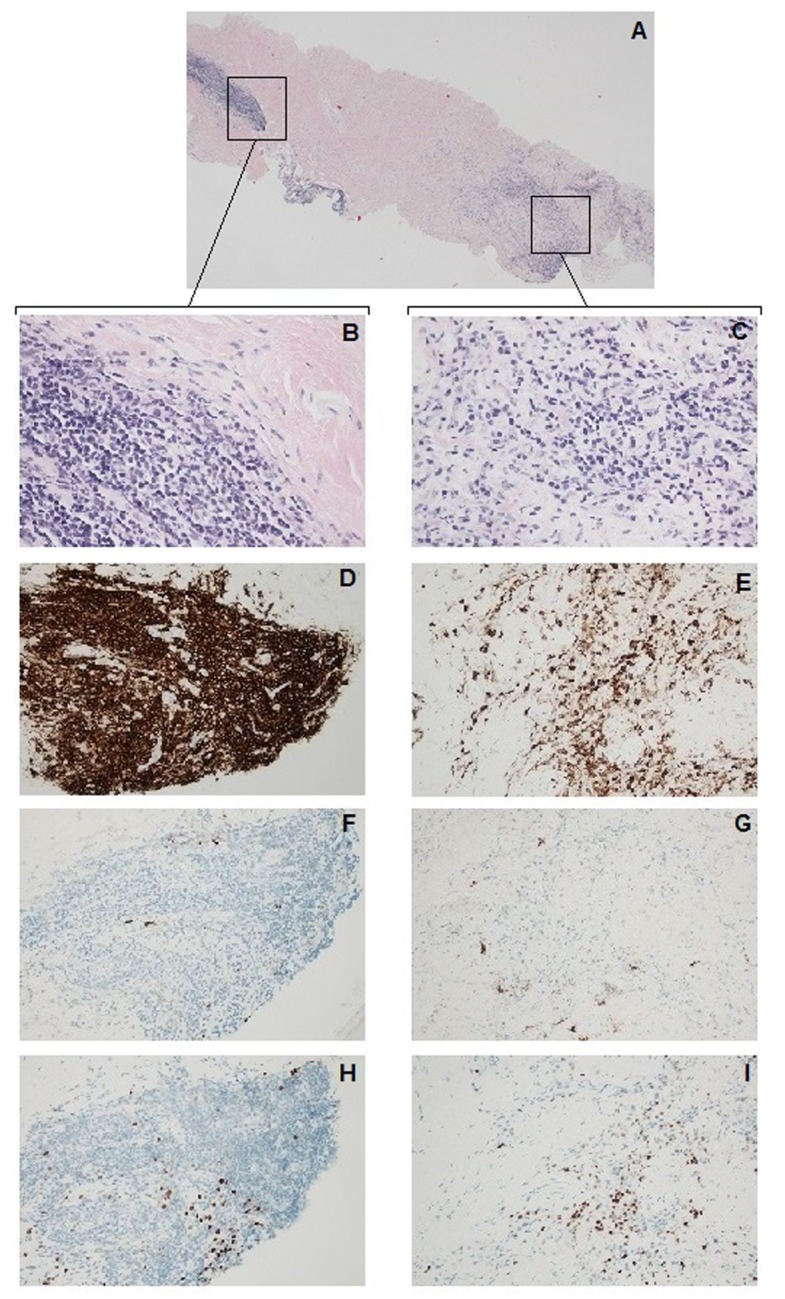
Right pulmonary artery biopsy. Histologic examination (hematoxylin and eosin stain) of the right pulmonary artery biopsy revealed fibrotic connective tissue **(A)** infiltrated by inflammatory cells consisting of lymphocytes and macrophages **(B,C)**. Immunohistochemistry was carried out for the leukocyte common antigen CD45 **(D,E)**, immunoglobulin subclass IgG4 **(F,J)** and a lymphocyte specific transcriptional factor: multiple MUM1 **(H,I)**. Total magnification of 40× **(A)** and 400× **(B–I)**.

Pulmonary endarterectomy was attempted 7 months later, during which, loose fibrous tissues were observed around the aorta, main pulmonary artery and superior vena cava. The right pulmonary artery appeared stiff and cryosection of the artery showed fibrosis and chronic inflammation. In light of the new findings, which were not compatible with CTEPH, pulmonary endarterectomy was aborted. Cabergoline treatment was discontinued immediately after the attempted endarterectomy as it was suspected to have caused the fibrotic changes and anticoagulation therapy was discontinued a few days later. Histologic examination of the right pulmonary artery biopsy (**Figure 2**) confirmed the cryosection results. Immunohistochemical screening for overexpression of MUM1, a lymphocyte-specific transcription factor associated with a wide range of hematolymphoid neoplasms (Natkunam et al., 2001), and IgG4, which can be associated with idiopathic fibro-inflammatory disorders (Rossi et al., 2016), were negative. Subsequent bronchoscopy showed no endobronchial abnormalities. Endobronchial biopsy and transbronchial needle aspiration biopsy showed nonspecific changes with no evidence of malignancy or granulomatous inflammation. Repeat HRCT showed pleural thickening and no evidence of retroperitoneal fibrosis. Repeat PET-CT showed hypermetabolic activity around the lung hila and aortic arch and in the right lung. Based on the clinical findings, histopathology and after ruling out other possible diseases, a diagnosis of mediastinal and pleuropulmonary fibrosis was made.

In the year that followed, the patient was monitored biweekly. Her prolactin level remained within normal range without dopamine agonist treatment. She received immunosuppressive therapy, prednisolone, methotrexate and mycophenolate mofetil, as well as pulmonary hypertension treatment, sildenafil, spironolactone and bumetanide. However, her symptoms deteriorated despite radiological signs of disease regression. She was hospitalized several times due to hemoptysis and opportunistic infections. Endovascular stenting of the right pulmonary artery was considered unsafe due to a high risk of arterial rupture. Double lung transplantation was assessed to be technically feasible and after much deliberation and detailed discussions at multidisciplinary meetings, it was considered the only option and the patient was admitted for the procedure. Upon sternotomy, the right lung appeared fibrotic and atelectic. It was adhered to the inner chest wall and appeared to be receiving part of its blood supply from the chest wall. Sadly, although the donor lungs were transplanted, the patient died on the operating table due to uncontainable bleeding from the chest wall and intercostal arteries.

## Discussion

We have presented an unusual complex case of pulmonary hypertension. It remains unclear whether this is a case of pulmonary hypertension complicating mediastinal fibrosis or multi-organ affection with concurrent fibrotic changes in the mediastinum, pulmonary artery, right lung, and pleura. It is debatable whether there is an association with the ergot-derived dopamine agonists, cabergoline and bromocriptine, that the patient had received.

Pulmonary hypertension complicating mediastinal fibrosis shares many similarities with proximal CTEPH including perfusion defects on CTPA and V/Q scintigraphy (Seferian et al., 2015), both of which have a high sensitivity for the diagnosis of CTEPH (Galie et al., 2016). The most common pattern of mediastinal fibrosis on CT is large, localized soft tissue masses with or without calcifications, while diffuse homogeneous soft tissue processes are less common (Sherrick et al., 1994; Worrell et al., 2007). Hence, the lack of clear radiological evidence of mediastinal masses, lymphadenopathy and/or extrinsic compression and encasement of the pulmonary arteries by neighboring tissues (Seferian et al., 2015) and the nonspecific lung parenchymal affection in this patient led to the incorrect diagnosis of CTEPH. Due to the variable etiologies and clinical presentations, there is no standard therapy for mediastinal fibrosis. Pharmacology treatment is often ineffective. However, there are reports of successful treatment with corticosteroids and mycophenolate mofetil (Lal et al., 2005; Ikeda et al., 2007; Witschi et al., 2009). Pulmonary hypertension related to mediastinal fibrosis is associated with a poor prognosis. Invasive interventions include endovascular stenting and bypassing or reconstructing an obstructed pulmonary artery (Brown et al., 2009; Seferian et al., 2015). In the current patient case, as pharmacological treatment was unsuccessful and direct manipulation of the right pulmonary artery was associated with a high risk of artery rupture due to the extensive fibrotic changes, lung transplantation was considered the only option. To the best of our knowledge, this is the first described case of attempted lung transplantation in a patient with mediastinal fibrosis, which, regrettably, was unsuccessful.

Ergot-derived dopamine agonists, such as cabergoline, pergolide and bromocriptine, can induce cardiac valve fibrosis and less frequently, non-cardiac fibrotic changes, such as pleuropulmonary and retroperitoneal fibrosis, especially in patients on long-term high-dose treatment for Parkinson’s disease, usually a cumulative dose >2000 mg in the case of cabergoline. The underlying mechanism is attributed to their interaction with serotonin receptors (Schade et al., 2007; Zanettini et al., 2007; Andersohn and Garbe, 2009). Low-dose treatment for hyperprolactinemia is not associated with an increased risk of clinically significant cardiac valvulopathy. However, mild to moderate tricuspid regurgitation and subclinical fibrotic lesions appear to be more prevalent during cabergoline and bromocriptine treatment and pulmonary arterial pressure, obtained on transthoracic echocardiography, was reported to be higher, but within normal range, in patients receiving bromocriptine (Elenkova et al., 2012; De Vecchis et al., 2013).

Several previous case reports that suggest a possible link between low-dose ergot-derived dopamine agonist treatment and clinically significant fibrotic changes are relevant to summarize here. Severe mitral regurgitation caused by cabergoline therapy (cumulative dose: ∼250 mg) was reported in a patient with hyperprolactinemia (Cawood et al., 2009). After daily low-dose cabergoline treatment (cumulative dose: ∼180 mg), a patient with acromegaly developed severe tricuspid regurgitation and dilated right ventricle with impaired function (Izgi et al., 2010). In a Parkinson patient, enlarged pulmonary artery, severe pulmonary hypertension, pleural effusion and mediastinal lymphadenopathy occurred after just one year of cabergoline treatment (cumulative dose: ∼730 mg) (Haro-Estarriol et al., 2009). Finally, constrictive pericarditis was diagnosed in a hyperprolactinemia patient treated with bromocriptine (cumulative dose: 14600 mg) and cabergoline (cumulative dose: 320 mg) (Londahl et al., 2008).These cases illustrate individual variations in susceptibility to the fibrotic side effects of ergot-derived dopamine agonists.

Drug-induced pulmonary hypertension is well-recognized. The best documented are the sympathomimetic anorexigens, aminorex, benfluorex and other fenfluramine derivatives, that have direct and indirect serotonergic effects (Seferian et al., 2013). However, there is no clear evidence of a causal relationship between ergot-derived dopamine agonists and pulmonary hypertension. Pergolide and methyseride, both of which are ergot-derivatives, are possibly associated with pulmonary hypertension (Egermayer et al., 1999; Seferian et al., 2013). Cabergoline and bromocriptine display agonist properties at several serotonin receptors, including 5-HT_1B_, 5-HT_2A_, and 5-HT_2B_ (bromocriptine is a 5-HT_2B_ antagonist, however) (Newman-Tancredi et al., 2002), all of which are implicated in the development of pulmonary hypertension. 5-HT_1B_ receptors mediate serotonin-induced pulmonary vasoconstriction and vascular smooth muscle hypertrophy and hyperplasia, 5-HT_2A_ receptors induce pulmonary arterial adventitial fibroblast proliferation and 5-HT_2B_ receptors are involved in right ventricular fibrosis (MacLean and Dempsie, 2010; Janssen et al., 2015). Consistent with this, macroscopic and microscopic structural changes were observed in the right pulmonary artery of our patient suggesting that pulmonary hypertension was caused by direct pulmonary artery affection possibly in addition to extrinsic compression caused by mediastinal fibrosis.

## Concluding Remarks

It remains undetermined whether cabergoline and/or bromocriptine treatment was the cause of mediastinal fibrosis and pulmonary hypertension in this patient. Further investigations are required to elucidate whether there is an association between ergot-derived dopamine agonists and mediastinal fibrosis and pulmonary hypertension. In conclusion as CT and V/Q scintigraphy findings can occasionally be deceptive, physicians should be particularly aware of differential diagnoses in patients without obvious history of venous thromboembolism that are suspected of having chronic thromboembolic pulmonary hypertension.

## Ethics Statement

Our submission is a case report. Data was collected during the routine clinical care of the patient. Prior to her death, the patient had given written informed consent to participate in a research study conducted by our group that was approved by The National Committee on Health Research Ethics (reference M-2013-278-13), including permission to collect and publish clinical data in anonymized form. Confidentiality of the data has been ensured in accordance to the ICMJE guidelines.

## Author Contributions

SM and JC were involved in the care of the patient. JS, SM, and JC collected the data and all the authors (JS, US, JC, and SM) contributed to the interpretation of the data. JS drafted the manuscript and all the authors have revised it critically for important intellectual content and approved the final version of the manuscript and this submission.

## Conflict of Interest Statement

The authors declare that the research was conducted in the absence of any commercial or financial relationships that could be construed as a potential conflict of interest.

## Abbreviations

CI: cardiac index
CT: computed tomography
CTEPH: chronic thromboembolic pulmonary hypertension
CTPA: CT pulmonary angiography
DSA: digital subtraction angiography
EBUS: endobronchial ultrasound
FDG: fluorodeoxyglucose
FEV1: forced expiratory volume in the first second
FVC: forced vital capacity
HRCT: high resolution CT
IVC: inferior vena cava
MUM1: myeloma oncogene 1
NT-proBNP: terminal pro-B-type natriuretic peptide
PAPm: mean pulmonary arterial pressure
PAWP: pulmonary arterial wedge pressure
PET: positive emission tomography
PVR: pulmonary vascular resistance
TAPSE: tricuspid annular plane systolic excursion
V/Q: ventilation/perfusion.
